# Enhancement of Mechanical Properties of Multilayer Ceramic Capacitors through a BaTiO_3_/polydopamine Cover Layer

**DOI:** 10.3390/polym15194014

**Published:** 2023-10-07

**Authors:** Yong Park, Jung Jin Park, Kwan Soo Park, Yong Min Hong, Eun Jung Lee, Sang Ouk Kim, Jong Ho Lee

**Affiliations:** 1 MLCC Green Chip Lab, MLCC Development Team, Samsung Electro-Mechanics, Suwon-si 16674, Republic of Korea; ydragon.park@samsung.com (Y.P.); jungjin21.park@samsung.com (J.J.P.); ks8708.park@samsung.com (K.S.P.); ym1012.hong@samsung.com (Y.M.H.); eunjung2599.lee@samsung.com (E.J.L.); 2 Department of Materials Science and Engineering, National Creative Research Initiative Center for Multi-Dimensional Directed Nanoscale Assembly, Korea Advanced Institute of Science & Technology (KAIST), Daejeon 34141, Republic of Korea; sangouk.kim@kaist.ac.kr

**Keywords:** multilayer ceramic capacitor, BaTiO_3_, cover layer, polydopamine

## Abstract

To fabricate multilayer ceramic capacitors (MLCCs) that can withstand external impacts, technologies to achieve excellent adhesion and mechanical strength of the cover layer should be essentially developed. Low adhesion and strength of the cover layer can lead to delamination and cracks in the MLCC, respectively. In this study, we present a method for applying polydopamine (PDA), a mussel-inspired adhesive protein, for as robust cover layer on an MLCC. Barium titanate (BT) particles treated with PDA increase the dispersion stability of the BT/PDA slurry, preventing re-agglomeration of the particles and enhancing the adhesiveness and strength owing to the cohesive properties of PDA. Compared to the BT layer, the adhesion of the BT/PDA layer was significantly enhanced by 217%; consequently, the compression modulus of the BT/PDA cover layer increased by 29.4%. After firing, the N-doped graphitic PDA played an important role in producing an MLCC cover layer with increased hardness and toughness. Furthermore, the N-doped graphitic PDA with a hydrophobic surface forms tortuous moisture paths in the cover layer, preventing the degradation of insulation resistance of the MLCC.

## 1. Introduction

**Figure 1:**
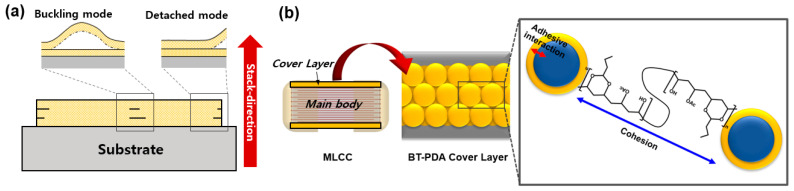
(**a**) Schematic of delamination mode of a cover layer. (**b**) BT/PDA composite structure in the cover layer of an MLCC.

A ceramic capacitor temporarily charges and discharges electricity. It regulates the current flow in a circuit and prevents electromagnetic interference. Multilayer ceramic capacitors (MLCC) consist of several single-layer capacitors stacked together in a package [1]. An MLCC consists of a body in which single-layer capacitors are stacked between the top and bottom cover layers (Figure 1). The mechanical properties of the cover layer are crucial for protecting the inner dielectric components of an MLCC. High strength and adhesiveness are required for the insulated cover layer of an MLCC. The poor interfacial adhesion properties of cover layers result in delamination in buckling and detachment modes (Figure 1a) [2]. These local interfacial defects remain after sintering and initiate crack propagation [1]. In addition, scratches, cracks, and other difficulties occur because of the low hardness and toughness of the cover layer [3]. Therefore, to address the chronic defects in MLCCs, improving the mechanical properties of the layers is essential.

Various studies have been conducted on poly(vinyl butyral) (PVB)-binder matrix systems to enhance the mechanical properties of ceramic composite layers before firing [4,5,6]. PVB-based composites are useful for fabricating functional films with deposited metal layers [5], and the rheological properties of PVB in barium titanate (BaTiO_3_, BT) suspensions contribute to slurry stability when used as a binder and dispersant [7,8]. In addition, PVB with a hydroxyl group is favorable for hydrogen bonding, which can be explained by its interactions with polymers, liquids, and particles in the BT slurry [6,8,9]. This interaction improves adhesion between the sheets, reducing the occurrence of de-lamination in the buckling and detachment modes [2,6]. Hence, it is important to enhance the adhesion of the material to the polymer composites.

The commercialization of MLCCs depends on various factors, such as their mechanical properties and moisture resistance, both before and after firing [10]. Graphene-added ceramic layers have excellent mechanical properties [11], chemical stability, and moisture barrier properties [12]. In addition, several studies have been conducted to improve strength and toughness [13].

Although composites with graphitic materials offer various benefits, they are difficult to adopt, owing to their low compatibility. Previous studies have shown that when graphene is embedded in a ceramic/polymer matrix, its aggregation leads to phase separation, degrading the mechanical properties of the composite [14].

In this study, we report a novel ceramic/polymer composite structure that satisfies both the pre- and post-firing requirements using polydopamine (PDA), which is a universal adhesive protein with both catechol and amine groups. Furthermore, PDA has attracted significant attention as a surface-modification technology that provides excellent adhesion to both organic and inorganic surfaces [15]. PDA is easily modified through one-step polymerization on the particle surface and has a polar functional group; therefore, it can be stably dispersed and adopted in various systems [9,15,16].

In BT/PDA system, the uniform PDA coating on the BT surface increased the interfacial adhesion and cohesion of the cover layer because of its interaction with the hydrophilic BT surface. Additionally, the chemical structure of PDA is highly compatible with the ceramic matrix [9,16]. Heat treatment with PDA induces structural changes in the N-doped graphitic carbon, including pentagons and heptagons [17]. After firing, graphitic PDA exhibits a plate-like structure (2-dimensional materials) with a hydrophobic surface and enhanced moisture barrier properties [12].

In this study, a new type of PDA-based cover layer for an MLCC is developed to improve mechanical, electrical, and moisture resistance. The functionalized PDA on the surface of BT formed hydrogen bonds with the composite (Figure 1b). Furthermore, PDA with catecholamine enhanced interlayer adhesion. The adhesion force and modulus of the BT/PDA layer increased by 217% (178 gf) and 29.4% (4.4 GPa), respectively, compared to those of the BT layer. The enhancement of the adhesive force and modulus strengthened the bonding force between the MLCC cover layer and the body; thus, interfacial defects, such as delamination, were substantially reduced. After firing, PDA was converted into an N-doped graphitic structure through subsequent pyrolysis, leading to high hardness and toughness of the cover layer. Owing to the N-doped PDA, the hardness and toughness of the cover layer were enhanced by 13.6% and 172%, respectively. The structural modification of PDA after sintering reduces defects, such as scratches and cracks, and enhances the stability and reliability of an MLCC, such as insulation resistance (IR) degradation in humid environments. 

## 2. Materials and Methods

### 2.1. Materials

BaTiO_3_ particles, binders, plasticizers, and dispersants were obtained from Samsung Electromechanics (Seoul, Republic of Korea). Dopamine (DA) hydrochloride and hydrochloric acid (HCl 37%) were purchased from Sigma-Aldrich (Burlington, MA, USA).

### 2.2. Fabrication of BT and BT/PDA Cover Layer

**Figure 2:**
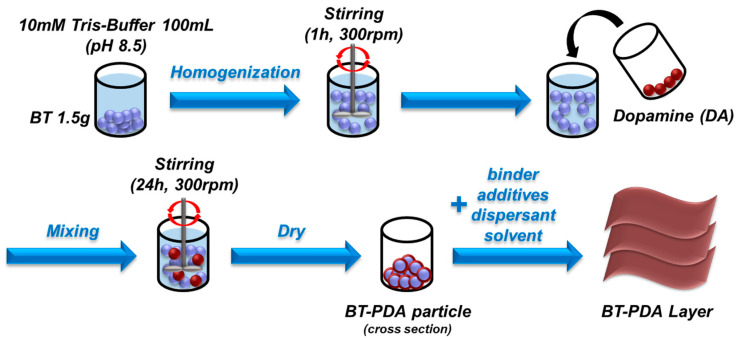
Schematic of the fabrication process of the BT/PDA cover layer.

BaTiO_3_ (80 nm) was added to 100 mL of a 10 mM Tris(hydroxymethyl)aminomethane solution (Tris-buffer, pH 8.5) for 1 h at 300 rpm. DA hydrochloride was then added to the homogenized BT solution and mixed for 24 h at 300 rpm. The DA-treated BT solution was washed 2 times with deionized water using a centrifuge and was then washed with ethanol. To obtain the BT/PDA particles, the solution was dried in an oven for 24 h at 100 °C. The binder solution was fabricated using PVB resin and a mixture of ethanol and toluene. The binder solution was sonicated for 2 h to achieve the target viscosity. The BT/PDA slurry was prepared by mixing the BT/PDA particles, binder solution, additives, dispersant, and solvent. A BT/PDA layer of 20 μm thickness was fabricated on the polyethylene terephthalate (PET) film with a doctor blade (Figure 2). The preparation of the BT layer followed a similar protocol as that used in the preparation of BT/PDA with the exception of PDA.

### 2.3. Surface Analysis

High-resolution transmission electron microscopy (HRTEM) images were acquired using a Talos F200X instrument. The chemical structure was analyzed using X-ray photoelectron spectroscopy (XPS, K-alpha, Thermo Fisher, Seoul, Republic of Korea).

### 2.4. Characteristics of Dielectric Sheets for the Cover Layer

Atomic force microscopy (AFM, XE-100, Park Systems, Suwon, Republic of Korea) was performed in contact mode to measure the adhesion force. The Si tip coating was then treated with a BT/PDA slurry. A BT layer was fabricated using the BT/PDA slurry on a SiO_2_ substrate. Force–distance (F–D) curves were measured with a force of 10 nN at ten random positions. The BT substrate was prepared in the same manner as the BT/PDA substrate, with the exception of PDA. The ball probe tack test was performed using texture analysis (TXA, YEONJIN S-TECH) to compare the relative adhesion (acrylic ball and BT, acrylic ball and BT/PDA). The experiment was conducted for 60 s under a load of 500 gf. The fabrication of the BT and BT/PDA substrates followed the method described for the F–D curve measurement. Dynamic mechanical analysis (DMA, TA Instruments, New Castle, DE, USA) was performed by measuring the strain and stress. Each specimen was pulled at a rate of 0.1 mm/s, and the tensile modulus was calculated from the initial slope of the elastic regime. The compression moduli were measured using a nanoindentation test system (iNano Indenter; KLA-Tencor, Kedah, Malaysia). The modulus was examined at a force limit of 50 mN and a depth of 500 nm.

### 2.5. Characteristics and Performances after Firing 

High-resolution thermogravimetric analysis (TGA) was conducted using TG209 F1 Libra by heating the sample up to 1000 °C at a rate of 3 °C/min (Hanau, Germany). Samples of each BT/PDA layer before and after firing were prepared for Raman spectra analysis. The preparation of the BT layer followed a similar protocol as that used in the preparation of BT/PDA with the exception of PDA. Raman spectroscopy was performed using a LabRAM HR Evolution Visible_NIR with a 514 nm laser (HORIBA, Kyoto, Japan). Image measurements of the cover layer were performed using optical microscopy (OM, Keyence Co., Ltd., Osaka, Japan) in the dark field (DF) mode at 50× magnification. The cover layer was then peeled off using a pull-off tester (Autonics, Busan, Republic of Korea). After the tape was attached to the cover layer by rolling it five times, it was removed at a detachment speed of 2000 mm/s. The grain size of the cover layer was observed using field-emission transmission electron microscopy (FE-TEM, Hitachi, Tokyo, Japan). Energy dispersive spectrometry (EDS) mapping was performed to confirm the elemental distribution and content. The hardness and toughness were measured for 5 s with a micro indenter (Micro Combi Tester, CSM Instruments, SA, Peuseux, Switzerland) under loads of 10 and 500 gf, respectively. The IR degradation was obtained by measuring in 1-step using a reliability testing chamber. BT and BT/PDA MLCCs were prepared on the substrate for testing, respectively. Next, moisture resistance reliability was measured for 8 h at a voltage of 3.75 V, a humidity of 85%, and a temperature of 85 °C.

## 3. Results and Discussion

The insulating layer that forms the cover layer of an MLCC requires high strength and adhesion. A cover layer with low adhesion causes an increase in delamination, and low-strength characteristics cause problems such as low breakdown voltage, cracks, and scratches. We developed a cover layer with high adhesion and strength for an MLCC by introducing PDA.

### 3.1. Surface Characteristics

**Figure 3:**
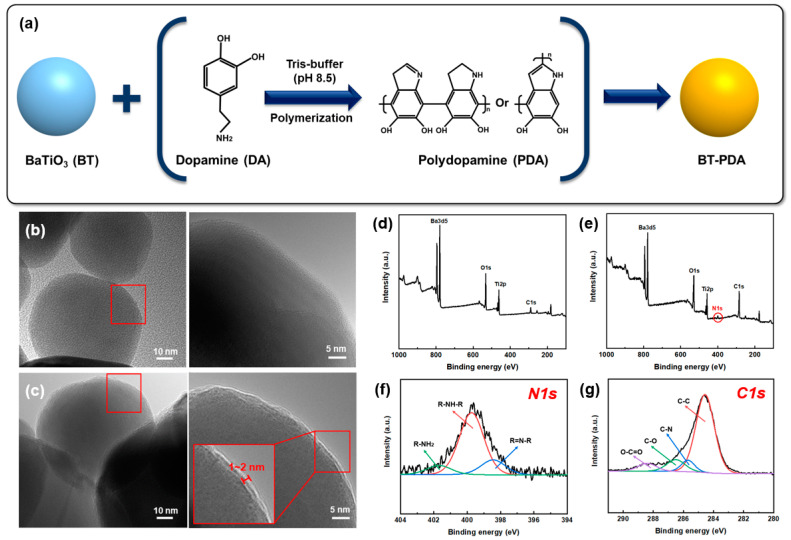
Surface analysis of BT and PDA-treated BT. (**a**) Self-polymerization mechanism of DA. HR-TEM image of the (**b**) BT and (**c**) BT/PDA particles. XPS spectra of (**d**) BT and (**e**) BT/PDA. High-resolution (**f**) N1s and (**g**) C1s spectra of BT/PDA surfaces.

Figure 3a shows that the BT particles were treated with DA to form BT/PDA particles. Various research hypotheses have been proposed regarding the formation reaction mechanism for the surface modification of PDA. Quinone is formed by the oxidation of DA, and the 5,6-dihydroxyindole (DHI) intermediate is formed by an intramolecular cyclization reaction. Further, DHI has been reported to form PDA via oxidation and polymerization [15]. The DA-treated BT particles formed BT/PDA particles via self-polymerization at pH 8.5.

Figure 3b,c show HR-TEM images of the BT and BT/PDA nanoparticles, respectively. The BT surface in the pristine state was smooth, and the PDA coated on the BT particles formed a distinct rough surface layer [9]. The measured PDA coating layer formed a uniform thickness of approximately 2 nm, which was different from the bare BT surface.

The adhesion of PDA is achieved by various amine or catechol groups. Metal oxide particles such as iron oxide and TiO_2_ are bonded by the amine groups of PDA [6,18]. Therefore, in the case of BT particles, it is expected that the amine group of PDA is connected to the particle surface. Chemical analyses of BT and BT/PDA were performed using XPS. The nitrogen component was observed only in BT/PDA and not in BT because it originates from PDA (Figure 3d–g). The high-resolution N 1s spectrum of BT/PDA shows the coexistence of primary (R-NH_2_), secondary (R-NH-R), and tertiary (R=N-R) amines, which is a proposed PDA structural model (Figure 3f). The primary amine exhibited a peak at 401.9 eV, which corresponded to DA, and the secondary amine exhibited a peak at 399.9 eV, corresponding to the intermediate or PDA. The DHI intermediate and 5,6-indolequinone exhibited a tertiary amine peak at 395.5 eV. Thus, the covalent polymerization of 5,6-indolequinone and the noncovalent self-polymerization of the intermediate are consistent with studies demonstrating the existence of PDA [19,20]. In addition, the two peaks of BT/PDA C1s, C–N (285.5 eV) and O-C=O (288.5 eV), are evidence of the PDA coating on the BT surface (Figure 3g) [9].

### 3.2. Properties of Dielectric Sheets for the Cover Layer

**Figure 4:**
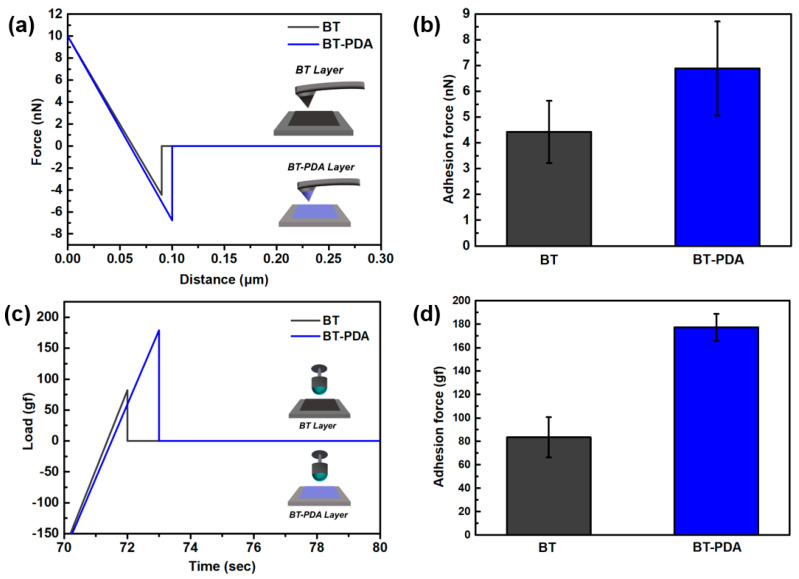
Adhesion force of the BT and BT/PDA layers. (**a**) F−D curves of the BT−BT and BT/PDA−BT/PDA layers. (**b**) Adhesion force values calculated from the F−D curves in (**a**). (**c**) Ball probe tack test of the BT and BT/PDA layers. (**d**) Adhesion forces calculated from the load vs. time curve in (**c**).

We conducted molecular-level adhesion analysis to confirm the interlayer interaction by AFM. After coating the BT and BT/PDA slurries on the Si tip and SiO_2_ substrate surface, the cantilever was bent to compare the adhesive strength through contact between the tip and substrate (Figure 4a). The F–D curve represents the physicochemical interactions between the substrate surface and the tip. The adhesion force calculated from the F–D curve was quantified and is shown as a bar graph. The measured F–D curve shows that the adhesion force between the BT/PDA and BT/PDA-coated tips (7 nN) was stronger than that between BT and the BT-coated tip (4.5 nN) (Figure 4b). Compared to BT, BT/PDA adhesion showed a significant increase of 55.5%.

TXA was performed to evaluate the debonding resistance before and after PDA treatment of the substrate. When the ball retracted from the substrate after physical contact, the adhesion force was calculated as an attractive force (Figure 4c). The debonding resistance results from the wetting of the ball and substrates, and is quantified by the peel force (Figure 4d).

The average adhesion force between the BT layer and ball was 82 gf, and the average adhesive force between the BT/PDA and the ball was 178 gf. BT/PDA exhibited a significant adhesion enhancement of 217%, which is larger than that of BT. The binder system in the slurry improved interlayer adhesion owing to hydrogen bonding with PDA, thus forming a strong bonding structure [6]. It has been demonstrated that PDA with the catechol group contributes to higher interlayer interaction in the composite as well as cohesion [15].

The AFM and TXA analyses confirmed adhesion enhancement resulting from surface interactions with PDA. To stack a cover layer with uniform thickness, minimizing the deformation caused by heat and pressure is necessary. A temperature higher than the glass transition temperature (Tg) is required for interfacial adhesion of the cover layer, and proceeding in the elastic region to reduce stacking deformation is preferable.

**Figure 5:**
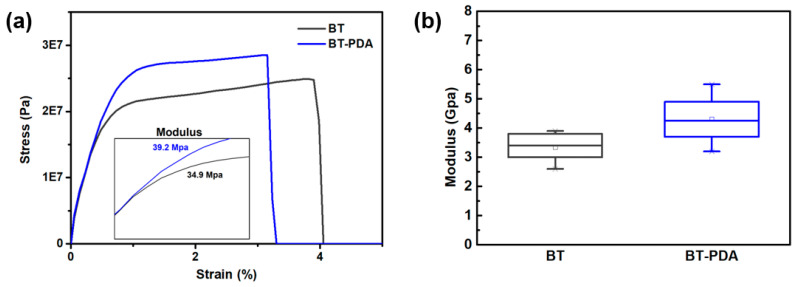
Moduli of the BT and BT/PDA layers. (**a**) Stress–strain curves of the BT and BT/PDA layers. The inset shows the tensile modulus values of the BT and BT/PDA layers. (**b**) Compression moduli of the BT and BT/PDA layers.

The tensile moduli of the BT and BT/PDA layers were measured using DMA. The compression modulus was determined using a nanoindenter because stacking deformation was affected by vertical stress. The tensile modulus of BT/PDA was 39.2 MPa, that is, 12.3% higher than that of BT (34.9 MPa). In addition, the compression modulus of BT/PDA increased by 29.4% (4.4 GPa) compared to that of BT (3.4 GPa). Figure 5a,b show the trends of modulus and strength enhancements in the BT/PDA layer under the same strain. Therefore, with the introduction of PDA, stacking deformation of the cover layer was reduced by improving the modulus. According to this analysis, the addition of PDA increased the modulus through interactions in the dielectric composites [21]. The formation of a hydrogen bond between the hydroxyl groups (–OH) of PDA and PVB forms a composite cover layer with high mechanical stability [6].

The modulus of the BT/PDA layer exhibited significant improvements compared with that of pristine BT. The BT/PDA layer formed hydrogen bonds via the interaction between the PVB binder and the catechol group of PDA. A positive impact of hydrogen bonding between PDA and PVB on the mechanical properties was observed.

### 3.3. Structure of PDA after Firing

The cover layer was produced using a firing two-step profile. The manufacturing process of an MLCC involves mixing a ceramic powder and binder, which is then molded into a sheet shape. Typically, an organic material is utilized as a molding binder, and the removal of the binder through pyrolysis is referred to as bakeout. In the subsequent firing process, densification by bonding between BT particles is called sintering [1]. 

The elimination of organic components was achieved by subjecting it to a decomposition process in a furnace under an Ar atmosphere at a temperature of 340 °C for 2 h. Subsequently, the temperature was increased under a N_2_ atmosphere at a rate of 10 °C/min to 1000 °C, and sintering was performed by firing the material for 1 h. Firing is a crucial process that involves the manipulation of various critical parameters, including temperature, atmosphere, and heating rate, to achieve optimal MLCC characteristics.

**Figure 6:**
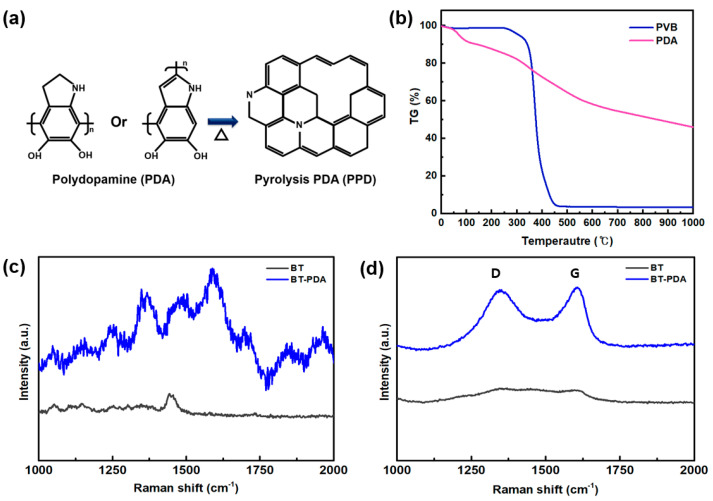
Chemical structure analysis of the BT/PDA layer after firing. (**a**) Chemical structure conversion during firing. (**b**) TGA results for the BT and BT/PDA layers. Raman spectra of the BT and BT/PDA layers (**c**) before and (**d**) after firing.

In contrast to the binders that are typically eliminated prior to sintering, it is widely acknowledged that PDA persists following structural modification. It is hypothesized that the structural transformation of PDA significantly affects its mechanical characteristics after firing. Specifically, PDA, which comprises crosslinked indolequinone units, undergoes graphitization via heat treatment to form pyrolysis polydopamine (PPD) (Figure 6a) [17].

To verify the weight alteration of the cover layer, TGA was conducted. The weight reductions in the primary organic constituents in the cover layer, PVB, and PDA were compared. The results (Figure 6b) suggest that the graphitic structure was converted from PDA when subjected to a temperature of 1000 °C. Conversely, the amount of PVB was a significant decrease in weight, approaching zero, when exposed to temperatures between 400 and 500 °C [22]. In the case of PDA, it was confirmed that carbon was not decomposed and still remained even at 1000 °C. Most of the PVB bonds were thermally decomposed through exothermic and steam carbon reactions and were removed as CO, CO_2_, and carbon compounds [23]. Most PDA was removed by increasing the temperature; however, in the case of crosslinked PDA, it was expected to remain on the cover layer through partial graphitization [24].

We investigated the geometric alterations before and after firing using Raman spectroscopy to validate the structural changes in PDA. The graphitization of the carbon materials was expressed as the I_D_/I_G_ ratio to quantify the extent of defects. In Figure 6c, the Raman spectra of BT and BT/PDA before firing show no signals, indicating the graphitization of carbon. Following the fire, D band (disordered carbon) and G (graphite carbon) peaks at 1350 cm^−1^ and 1600 cm^−1^, respectively, were visible in PDA (Figure 6d). The I_D_/I_G_ (=0.9) ratio of BT/PDA indicates that PDA was converted into a graphite structure with excellent properties [17]. However, no structural change peaks were observed for BT after firing. 

Therefore, the structural changes in BT/PDA after heat treatment were caused by PDA. However, no structural change peaks were observed for BT after firing. Therefore, the structural changes in BT/PDA after the heat treatment were caused by PDA. These changes prove that PDA was gradually converted from an amorphous state to a graphitic structure.

### 3.4. Performances of MLCC

We fabricated three types of samples with different concentrations of PDA in the cover layer, that is, 0, 0.5, and 1 wt%, as indicated by the abbreviations BT, PDA0.5, and PDA1, respectively. The agglomeration between PDAs was caused by the increased content above 1.0 wt%, resulting in strong interactions among residual PDA.

**Figure 7:**
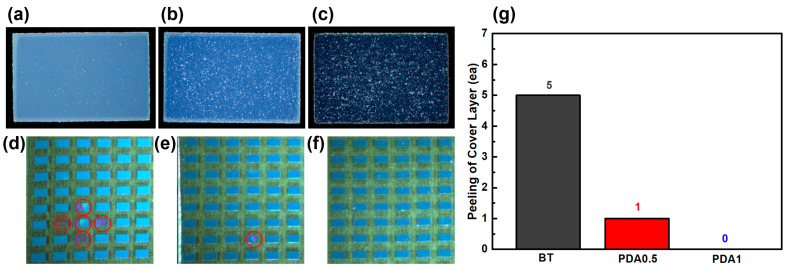
Surface of the (**a**) BT, (**b**) PDA0.5, and (**c**) PDA1 cover layers for an MLCC. Surfaces of the (**d**) BT, (**e**) PDA0.5, and (**f**) PDA1 cover layers for an MLCC after the pull-off test. (**g**) Peeling of the cover layer for BT, PDA0.5, and PDA1. The red circles show MLCC with the cover layer peeled off and the inner layer exposed.

The performance of the BT/PDA cover layer was evaluated for an MLCC, as shown in Figure 7. As the PDA content increased, the hues became increasingly darker. The dark-brown color of PDA combined with the BT results in a blended color (Figure 7a–c). A pull-off test was conducted to confirm the interfacial adhesion between the cover layer and the main body. Further, BT, PDA0.5, and PDA1 exhibited 5, 1, and 0 cover peeling, respectively, proving that the addition of PDA enhanced the interfacial bonding force between the cover and body (Figure 7d–g). 

EDS was used to examine the elemental composition of the BT interface using TEM-line profiling after firing (Table 1, Figure S1). The O, C, and N contents of BT and PDA1 are also listed in Table 1. The contents of O and C in BT and PDA1 were comparable; however, a substantial difference in the content of N in PDA1 (2.73%) compared to that in BT (0%) was observed. These results confirm that after firing, PDA remained as an N-doped graphitic structure at the BT interface.

**Figure 8:**
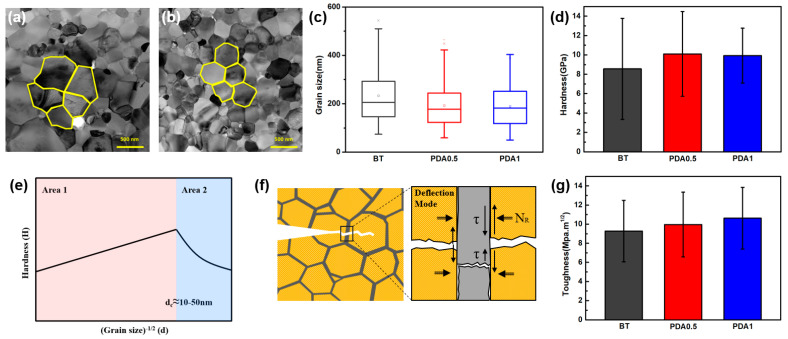
Mechanical properties of the BT and BT/PDA cover layers after firing. TEM images of (**a**) BT and (**b**) BT/PDA grains. (**c**) Grain sizes of BT, PDA0.5, and PDA1. (**d**) Hardness of BT, PDA0.5, and PDA1. (**e**) Schematic of Hall–Petch theory. (**f**) Schematic of the toughness theory mechanism of crack propagation. (**g**) Toughness of BT, PDA0.5, and PDA1.

When the grain size was confirmed by FE-TEM, a noticeable variation was observed between the BT and PDA1 grains (Figure 8a,b and Figure S2). Figure 8c shows a graph that compares the grain sizes of BT, PDA0.5, and PDA1 after firing. As the PDA content increased, grain size and variance decreased. After sintering, a partially N-doped graphitic structure exists at the grain interface, which inhibits grain growth [25]. The sintering process was conducted through the formation of necks between particles, and graphitic PDA, partially present in the cover layer, was used as a grain growth inhibitor [25]. In particular, the partially graphitic PDA present at the grain boundaries delayed the necking of BT, resulting in a smaller grain size in the cover layer. At the final sintering temperature, the pore is removed and densified because of the sufficient energy for sintering.

The introduction of the N-doped graphitic structure derived from PDA resulted in a decrease in grain size, which is representative of the behavior of harder materials. The hardness values of BT, PDA0.5, and PDA1 were 8.8, 10.1, and 10.0 GPa, respectively (Figure 8d).

The average values of PDA0.5 and PDA1 were equivalent; however, the decrease in variance observed in PDA1 suggests a more homogeneous distribution of grain size, resulting from the presence of graphitic PDA. Figure 8e shows the correlation between the grain size and hardness [26]. We focused on Area 1 because the grain size of the measured cover layer was larger than *d* > *d*c (critical grain size, 10–50 nm). The influence of hardness on the grain size in Area 1 is described by the Hall–Petch equation (Equation (1)) [26].

(1) $$H=H_{i}+kd^{-1/2}$$

where *H*, *Hi*, *k*, and *d* are the hardness, lattice friction stress, Hall–Petch constant, and grain size, respectively. The reduction in the grain size proportionally increased the volume of the grain boundaries. This, consequently, causes dislocation pinning. It is widely accepted that the critical factor responsible for enhancing strength is dislocation pinning, which results from an increase in grain boundaries [27]. Furthermore, for compressive and tensile stresses, the significant friction in BT ceramics with reduced grain size contributes to increased hardness [28].

Crack propagation in ceramic composites results in fatal defects, emphasizing the importance of a high-toughness cover layer. We presented a possible hypothesis to explain the crack propagation mechanism of the high-toughness cover layer. The propagation of local cracks in high-toughness composites is impeded by the elastic and strength properties of the heterogeneous materials. The strain resulting from cracking is resolved using Equation (2) [29].

(2) $$\alpha =\frac{E_{P}^{\prime }-E_{B}^{\prime }}{E_{P}^{\prime }+E_{B}^{\prime }}$$

where *α* represents the elastic mismatch parameters, and $E_{P}^{\prime }$ and $E_{B}^{\prime }$ represent Young’s moduli of graphitic PDA and BT, respectively. Because the sum of the moduli of the denominator has a positive value, *α* is determined by the difference between the relative values of $E_{P}^{\prime }$ and $E_{B}^{\prime }$ in the numerator. The commonly known modulus of the graphite structure is ≈1.1 TPa [30,31]. Furthermore, the difference in stiffness between materials is a crucial factor in describing the crack propagation mechanism. The deflection ratio of BT and graphitic PDA can be expressed as *Γ_BT_/Γ_PDA_*, where *Γx* is the fracture energy release rate of *x* component. *Γ_BT_/Γ_PDA_* is smaller than that of the homogeneous BT phase because of the high fracture energy of graphitic PDA. Hence, the cover layer with PDA exhibits superior quality, which results in the postponement of fracture and redirection of crack propagation [29].

Crack propagation and deflection can be described by the shear stress and force along the interface between materials with different moduli and internal frictions. The toughness of the cover layer can be explained by an interface-dependent mechanism [29].

*τ_failure_* = *τ_debond_* + μ*_i_N_R_* (3)

*τ_sliding_* = μ*N_R_* (4)

where *τ_failure_*, *τ_debond_*, *τ_sliding_*, μ, μ*_i_*, and *N_R_* are the failure stress, interface debond shear strength, frictional sliding stress, dynamic friction, internal friction coefficient, and residual clamping stress, respectively.

In the cover layer with graphitic PDA, crack propagation was first delayed by the redirection of the crack resulting from the low deflection ratio of graphitic PDA and BT (Equation (2)). Then, crack propagation requires the energy of interface failure, which is related to the interface debond shear strength (*τ_debond_*) and internal friction coefficient (μ) (Equation (3)). Subsequently, frictional sliding stress (*τ_sliding_*), as a function of dynamic friction, is required (Figure 8f and Equation (4)). This implies that multiple forms of energy are necessary for crack propagation in heterogeneous materials.

Figure 8g shows that the toughness values of BT, PDA0.5, and PDA1 were 9.3, 10.1, and 10.9 MPa·m^1/2^, respectively. Compared to BT and PDA1, the toughness of PDA1 was improved by 17.2%. The toughness gradually increased as the PDA content increased. This was influenced by the high elasticity and strength of PDA distributed among the BT particles. Crack propagation was delayed or stopped by the high fracture energy of PDA, which improved the toughness of the BT/PDA cover-layer structure.

**Figure 9:**
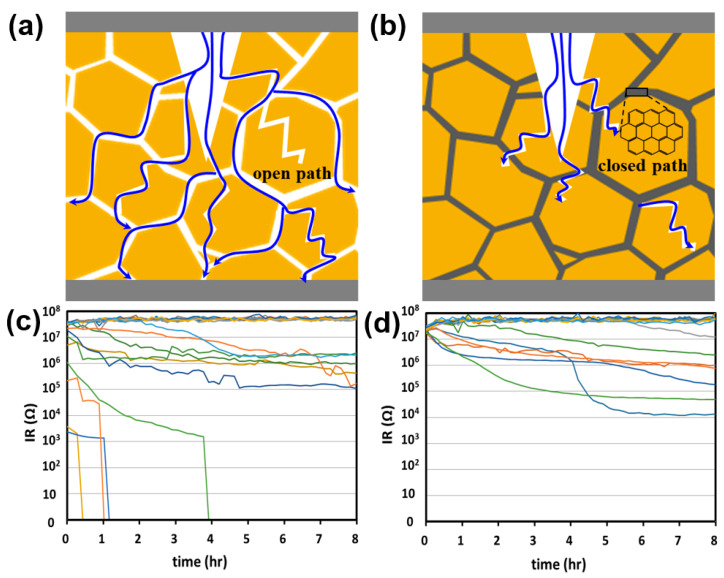
Moisture resistance. Schematic of (**a**) direct and (**b**) tortuous paths of the cover layer of an MLCC. IR degradation of (**c**) BT and (**d**) PDA1. The color lines represent each MLCC mounted on the board.

High-temperature and humidity tests were conducted to confirm the effect of PDA on the reliability of moisture resistance. Previous experiments have confirmed that the partial graphitic PDA of the cover layer can prevent moisture penetration [12]. The defect in the cover layer provided an open path; thus, the time required to reach the inner electrode and dielectric body was shortened (Figure 9a). In contrast, graphitic PDA at the BT interface acts as a moisture barrier to effectively prevent water penetration, leading to tortuous and closed moisture paths (Figure 9b) [12].

Figure 9c,d show the IR degradation results of the MLCC using the cover layers with BT and PDA1, respectively. For the BT sample, the MLCC chip exhibited an initial failure with a low IR value. On the other hand, for the MLCC with PDA1, the successful prevention of moisture penetration is attributed to an IR of ~10^7^. Tortuous and closed moisture paths resulting from graphitic PDA have been demonstrated to play a critical role in impeding or delaying the ingress of moisture into the internal electrode and dielectric body.

## 4. Conclusions

In summary, PDA was introduced into ceramic layers to enhance their mechanical and physical properties. PDA is a versatile adhesive material with mussel-inspired catecholamine groups that can adhere to both organic and inorganic materials. PDA was successfully coated onto the BT powder and evenly distributed in the PVB matrix. The coated PDA increased dispersibility by preventing the re-aggregation of the BT particles through adhesive interactions with BT. Additionally, the adhesion forces of PDA and PVB via hydrogen bonding improved the sheet strength and adhesion of the cover layer. This technology can reduce interlayer defects, such as delamination and deformation, through strong bonding between the MLCC cover layer and the body. During the firing process, the PDA structure was converted into N-doped graphitic PDA. N-doped graphitic PDA plays an important role in increasing toughness by reducing crack propagation and improving hardness, owing to grain size reduction after sintering. The enhancement of the hardness and toughness of the cover layer reduced structural defects such as cracks and scratches. In addition, the graphitic PDA in the cover layer formed tortuous paths, reducing the penetration of moisture into the interior. Compared with the BT layer, the initial IR failure—reduction of the BT/PDA cover layer indicates that graphitic PDA forms a barrier and becomes a closed path for moisture ingress. PDA was successfully incorporated into a BT cover layer to improve its pre- and post-firing properties. This study provides a new approach for developing chemically and mechanically robust MLCC cover layers.

## Figures and Tables

**Table 1:** EDS analysis of BT and PDA1.

Element	BT (at%)	PDA1 (at%)
O	43.22	42.83
C	1.52	3.12
N	0	2.73

## Data Availability

The data presented in this study are available upon request from the corresponding author.

## Supplementary Materials

The following supporting information can be downloaded at: https://www.mdpi.com/article/10.3390/polym15194014/s1, Figure S1: (a) TEM image of BT/PDA layer after firing. (b) N atomic percent by line profile. The line profile represents a higher atomic percent of N at the grain boundary; Figure S2: SEM image of (a) BT and (b) BT/PDA internal MLCC layer. Electrode connection of (c) BT and (d) BT/PDA internal MLCC layer. The internal MLCC layers of BT and BT/PDA are the same, except for the cover layer. The porosity of dielectric layer for the BT and BT/PDA was at the same level. When the perfect electrode connectivity is assumed as 100%, the calculated electrode connectivity of BT is 85%, and that of BT/PDA is 86%.
