# Supplementary material for: Enhancement of Mechanical Properties of Multilayer Ceramic Capacitors through a BaTiO3/polydopamine Cover Layer
Source: Polymers (Basel). 2023 Oct 7;15(19):4014. doi: 10.3390/polym15194014 (PMC10575367; doi:10.3390/polym15194014)
Supplement: Supplementary file 1 [file polymers-15-04014-s001.zip › polymers-2539916-supplementary.pdf]

Supplementary Information

Enhancement of Mechanical Properties of Multilayer Ceramic Capacitors through a $BaTiO_3$/polydopamine Cover Layer

Yong Park [1, †], Jung Jin Park [1, †], Kwan Soo Park [1], Yong Min Hong [1], Eun Jung Lee [1], Sang Ouk Kim [2], and Jong Ho Lee [1, *]

[1]MLCC Green Chip Lab, MLCC Development Team, Component Business Unit, Samsung Electro-Mechanics 150 Maeyoung-ro, Yeongtong-gu, Suwon-si, Gyeonggi-do, 16674, Republic of Korea

[2]National Creative Research Initiative Center for Multi-Dimensional Directed Nanoscale Assembly, Department of Materials Science and Engineering, KAIST, Daejeon 34141, Republic of Korea

*****Correspondence: jh205.lee@samsung.com

[†] These authors have contributed equally to this work.

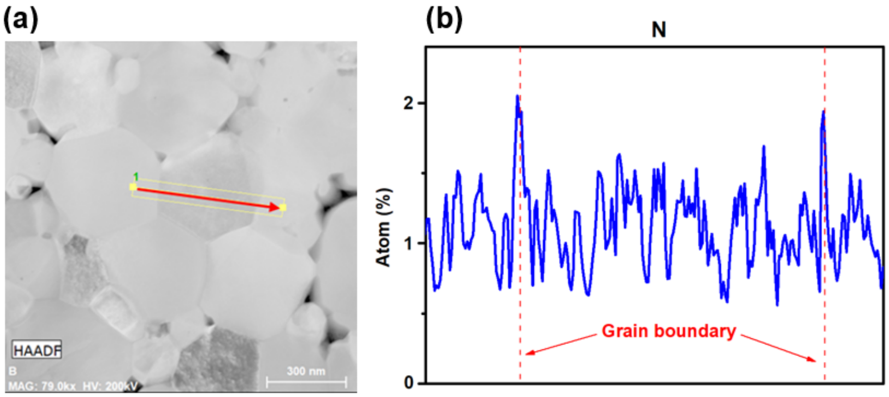

Figure S1. (a) TEM image of BT/PDA layer after firing. (b) N atomic percent by line profile. The line profile represents a higher atomic percent of N at the grain boundary.

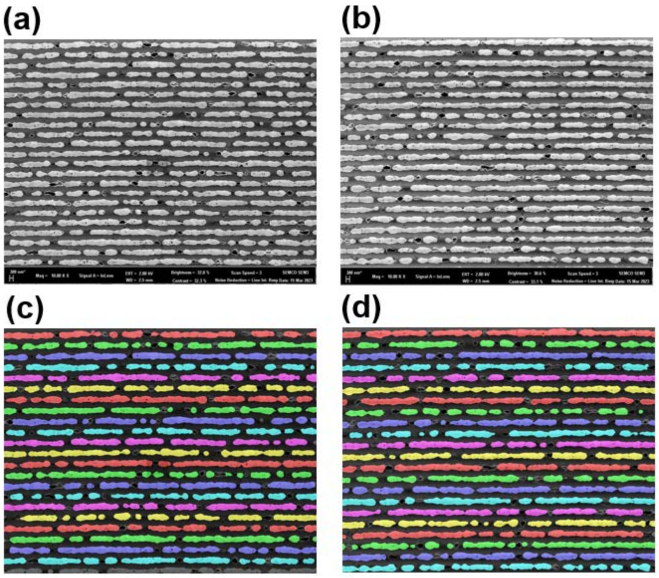

Figure S2. SEM image of (a) BT and (b) BT/PDA internal MLCC layer. Electrode connection of (c) BT and (d) BT/PDA internal MLCC layer. The internal MLCC layers of BT and BT/PDA are the same, except for the cover layer. The porosity of dielectric layer for the BT and BT/PDA was at the same level. When the perfect electrode connectivity is assumed as 100 %, the calculated electrode connectivity of BT is 85 %, and that of BT/PDA is 86 %.
